# A Practical Treatise on the Diseases Peculiar to Women, Illustrated by Cases Derived from Hospital and Private Practice

**Published:** 1845-06

**Authors:** 


Art. V.—*A Practical Treatise on the Diseases peculiar to Women, Illustrated by Cases derived from Hospital and Private Practice.* By Samuel Ashwell, M.D.; Member of the Royal College of Physicians, London, and Obstetric Physician and Lecturer to Guy's Hospital. First complete American from the last London edition. With notes by *Paul B. Goddard*, M.D., M.A.P.S., M.A.N.S., &c. Philadelphia: Lea & Blanchard. 1845. 8vo. pp. 520.

This treatise on the diseases of females is the fruit of twenty years' experience in that particular class of complaints. It is more than twenty years, says the author in his preface, since his attention was particularly directed to that subject, and for the greater part of that time his connexion with an extensive lying-in Charity, and his large private practice have afforded him opportunities which, he may justly say, "fall to the lot of few practitioners." His treatise was commenced years ago, and has all the completeness and maturity which time and protracted labor could impart to it. "My aim," he remarks, "has been to produce a treatise on female diseases, true, simple, and practical, which may form a safe and efficient guide to the elucidation and curative treatment of many of these intricate, rapidly-progressing, and dangerous maladies." He adds, "I have endeavored to write in a plain and perspicuous style, with scrupulous accuracy as to facts; and in reference to opinions and treatment, nothing is recommended, the probability or worth of which, my own experience has not confirmed."

These are important ends, and if the author has compassed them to the extent of his wishes, his work may safely be pronounced one of the first in the language. We believe this to be the case. We consider that the effort is a most successful one. We regard the volume before us as one of the best guides to which the practitioner could have recouse in this most perplexing order of diseases, and as such we cordially recommend it to the profession.

The complaints peculiar to women are arranged by Dr. Ashwell in three great divisions; namely: functional, organic, and those which are incident to the pregnant and puerperal states. He appropriates comparatively little space to the anatomy and physiology of his subject, but devotes himself strictly to pathology and treatment, which in a work for reference is a desirable feature.

The affection first considered is *chlorosis*, which he describes in three forms: as a mild and incipient, an inveterate and confirmed, and a complicated disease. The disease, he says, is occasionally met with in our own sex, of which he has seen one or two marked instances. It depends primarily on a morbid condition of the blood, which, in the female, secondarily affects the ovaries and uterus, by retarding their growth. It has nothing in it of an inflammatory character, but is most intimately connected with anæmia. Hence we are not to conclude that the lancet is called for in affections of this sort, although they may be attended with severe pain and a rapid pulse. These symptoms are rather indicative of irritability, "and demand narcotics, carminatives, and, at the most, counter-irritation, not bleeding, active purgatives, or spare diet."

Chlorosis never exists without more or less of amenorrhœa, but the latter may prevail for a length of time without the former, although in the end symptoms of chlorosis are pretty sure to be developed. The frequent concurrence of these diseases has led many to conclude that chlorosis and amenorrhœa are convertible terms; but this is a mistake; as already stated either one may bring on the other, and this is the relation which subsists between them. They frequently present themselves together in girls about the age of puberty, and if neglected lead to fatal consequences, the brain, digestive apparatus, or lungs becoming involved. It is hence the duty of the physician to admonish the parents of a danger which may be unsuspected, that by medicine, or a change of air and habits the malady may be checked in its incipient stage.

"The treatment of the most common form of chlorosis, namely, that accompanying puberty, may be regarded as the type of the treatment of all the others; embodying the principles, which, with greater or less modification, are universally applicable. It is here, at the very threshold of the disease, when its character is not understood, or when it is treated empirically, that the greatest error is committed. It is viewed as a local, not as a constitutional affection; and many are the individuals who have been sacrificed to the vain and ignorant attempt of prematurely establishing menstruation; mercury, drastic purgatives, and emmenagogues, having irretrievably destroyed the constitutional power and paved the way for phthisical disease.

"It is not my intention elaborately to comment upon certain great mistakes in the *physical education* of female youth. And yet, I must be excused, if I direct attention to the diet, air, exercise, and clothing of the sex. It will readily be granted, that if, in these particulars, there is extensive deviation from the dictates of nature and common sense, there must be a proportionate risk of debility and disease. In our own changeable climate, it behooves the guardian of female youth to be especially prudent; and I am one of those who think, that it is scarcely possible to study these matters too closely. If the national practices in these particulars could be changed—and the remark applies with great force to the middle and higher classes of society living in cities and towns—chlorosis, imperfect puberty, and amenorrhœa, would be uncommon, instead of being, as they now are, extremely prevalent diseases.

"Chlorosis is a rare affection in rural districts; where female youth are much in the open air, where it is not unfashionable to walk and run, and where it is not considered a gross violation of good breeding to sport and play with activity and vigor. Such girls acquire energy of system, each organ is developed, the blood is abundant and of excellent quality; nutrition is healthy, and puberty is attained without difficulty."

A morbid state of the blood lies at the basis of the disease and must be corrected by improving the digestive functions. A due evacuation of the bowels must be daily secured, and for this purpose Dr. Ashwell prefers aloes, rhubarb, the sulphate of soda and manna, to be aided if necessary by the hydrargyrum cum cretâ as an alterative. An injection of a pint of warm water into the rectum, two or three times a week, he also looks upon as a valuable measure for unloading the large intestines. Aromatics are advantageously combined with the laxatives, and, above all, iron at a particular stage of the disease is the remedy upon which our main reliance must be placed. But it cannot be resorted to without due preparation of the system; if the iron be given before the function of the digestive apparatus has been improved by purgatives, and while the tongue is still loaded and foul, aggravation of symptoms will be the consequence. This condition being obviated, and only the peculiar debility and pallor of chlorosis remaining, a single grain of the sulphate of iron, or even twice that quantity, may be most beneficially tried. Occasionally, as stated by our author, the effect is almost magical, and is manifest in proportion as it does not confine the bowels nor induce febrile heat. Dr. A. prefers to combine the iron with narcotics, and recommeds the following formula:

"Ferri ammon. ʒiss, extr. humuli, extr. papav. alb. ää gr. xv., ol. cassiæ m. xv., M. ft. pil. xxiv. Sumat. i. vel. ij. bis terve quotidie."

We agree with our author, that a most important addition to our remedial resources in this affection has been made in the *iodide of iron*, some interesting cures by which we have witnessed. He indicates in the following passage the circumstances under which it proves most beneficial, as well as a most eligible form in which to administer it:

"The iodide of iron has been extensively tried, both in hospital and private practice, and with undoubted success; especially when glandular enlargements and other indications of a strumous habit, have been associated with the chlorosis. I give it in the subjoined form.

"Ferri iodidi gr. xvi., tinct. columbæ vel. gent. c. ℥i., aquæ distillatæ ʒvii., ft. mist. Sumat coch. ii. magna, bis terve quotidie."

The next disease, *amenorrhœa*, is of much more frequent occurrence than the one just treated of. There are two principal forms of it: amenorrhœa of retention, and amenorrhœa of suppression. The author is very full on all the points connected with the pathology and treatment of this complaint, and indicates with clearness the various conditions which call for variety in the course of cure, such as congenital deficiency or diseases of the organs of generation, a too plethoric, or a too delicate and irritable state of the body, &c. In a case of recent and acute suppression in delicate, spare women, who are highly nervous and irritable, the following is his plan of treatment:

"General bleeding is inadmissible, nor are leeches usually advantageous: metastasis of the pain, but rarely its permanent removal may be produced by their application. Active purgatives are necessary, for the bowels are commonly loaded, and hard scybalous fæces long retained in the large bowels, excite and maintain painful irritation. A general warm bath at 96°; a warm mustard hip-bath or mustard pediluvia, may be advantageously employed. The following antispasmodic draught may be given every three or four hours, till the symptoms begin to subside.

℞ Liq. Ammon. Acet ʒii vel. iii.

Tinct. Castorei vel. Asafœtidæ ʒss. ad ʒj.

Pulv. Ipecac. C. gr. iv. vel. v.

Mist. Camph. ℥vii. M. ft. Haust.

"In addition, if pain be severe, a pill, containing two or three grains of camphor, and two grains of antimonial powder may be exhibited.

"Injections into the rectum sometimes produce an almost magical effect. Laudanum, asafœtida, and poppy syrup are employed for this purpose, (vide formulæ) and as it is necessary that they be retained for some time after their introduction, a piece of sponge or a knapkin, should be kept firmly and closely applied to the extremity of the bowel. When narcotic enemata are injected, the quantity should not exceed two or three ounces, as more will unnecessarily dilute the anodyne ingredient, and by distending the gut, induce expulsive action. The pain and spasm, in this form of acute suppression, are thus relieved, and menstruation does oftener recur during the immediate period, in this, than in the inflammatory species; but in neither can it be invariably expected. If, however, the treatment has fortunately re-established the discharge, every precaution ought to be employed to prevent the exposure of so susceptible a patient to any of those causes which might induce a relapse. It need scarcely be remarked, that an attack, either of inflammation or irritable suppression, is often the prelude to more permanent menstrual obstruction; and if month after month elapses, without the performance of the secretion, chronic suppression to be next treated of, will be the result."

In chronic suppression, the state of the system determines the character of the treatment, which must be antiphlogistic if plethora exist, and tonic and stimulant if the reverse. But there occur cases often in which complications make it necessary to modify the plan of cure. The following quotation from our author will give a good idea of the discrimination which is necessary to the successful management of this disorder:

"The six cases of complicated amenorrhœa were very interesting. In one, it was associated with chorea. The patient, after protracted treatment, was eventually cured by sulphate of zinc, and the injection of liq. ammoniæ into the vagina. In another, amenorrhœa was complicated with epilepsy. The medicine prescribed was ferri sulph. gr. i. pulv. digitalis gr. i pulv. myrrhæ gr. ij. mucil. Acaciæ q. s. fiat pillula ter die sumenda. It is worthy of remark, that these pills were persevered in for three weeks, without any injurious consequences from the use of the digitalis; a circumstance attributable, probably, to its combination with the iron. At this period, the catamenia appeared; and there has been no return of the fits. In a third case, hemiplegia was attendant on the amenorrhœa. This complication was tedious, and difficult to manage. At first, the mist. ferri c. was prescribed; afterwards, the sulphate of zinc; and an iodine liniment was well rubbed over the spine, night and morning. Menstruation was eventually established, and the patient regained the entire use of the side. In the fourth case, there was tænia with the amenorrhœa. In addition to the other remedies, the ol. terebinth. was curatively employed. In the fifth patient there was vicarious discharge from the mammæ, in conjunction with amenorrhœa; the mist. ferri c. was ordered, as well as the daily employment of the ammoniacal injection. The last patient had, in addition to the amenorrhœa, a peculiar nervous affection of one of her lower extremities which completely subsided when the catamenial function was, by appropriate remedies, healthily established."

Among emmenagogues Dr. A. speaks favorably of *electricity*, moderately applied, where the system is not plethoric, where pregnancy is not suspected, where, in a word, the disease is local, all constitutional symptoms having been subdued. Stimulating injections are also mentioned in encouraging terms.

"These were formerly much employed, and a variety of irritating ingredients entered into their composition; at present, as a vaginal enema, the Liq. Ammoniæ fort. in milk, is generally administered. I have often used it with success during the last twelve years, both in hospital and private practice. It rarely does good, if it is not attended and followed by a pungent sensation of heat, tingling, and some pain in the vagina. Its use should be commenced three days prior to the expected period: and the patient, after each injection, shoud apply a napkin to the vulva, so firmly as to cause the injected fluid to be retained for ten or fifteen minutes. It is not a safe remedy where there is uterine congestion. In two such cases dangerous inflammation of the cervix and upper part of the vagina followed its use. Where, however, uterine torpor is unaccompanied by congestion and acute irritation, the ammoniacal injection is often efficacious. Occasionally, like electricity, it produces menstruation at once, while, in some women, in common with the most approved remedies, it is without effect. The strong mustard hip-bath, used twice during the day, the patient remaining in it for nearly an hour each time, at a temperature of 96 or 98°, is an effectual auxiliary remedy."

He gives the following formula for the injection:

"℞ Liq. ammon. fort. ʒi. vel. ʒiss., lactis tepid. ℥xvj. M. ft. Injectio vaginalis. A third part to be passed into the vagina three times daily.

In the next chapter the author considers *vicarious menstruation.* This is often witnessed as hæmatemesis, hæmoptysis, or in a discharge of blood from the nipples, and at other times in the form of a severe salivation. Leucorrhœa is also vicarious, in some instances, of the sanguineous menstrual discharge. Dr. Ashwell has the following on that head:

"There is in health a secretion, exceedingly small in quantity, of colorless transparent mucus, poured out by the uterine lining membrane, for the purpose of lubricating the opposite surfaces of the organ, and preventing friction and adhesive inflammation. When excessive, constituting leucorrhœa, it is occasionally, and more frequently than blood, vicarious of menstruation. Strictly speaking, there is amenorrhœa, because a mucus, instead of a sanguinous secretion, is furnished by the minute extremities of the uterine arteries. But there is activity instead of torpor; and it will be found on inquiry, that all the symptoms denoting menstruation regularly appear, especially when this condition is vicarious of the catamenia at an early age.

"The disease is most common in delicate and susceptible girls, at the epoch of commencing menstruation. I have seen it also in weak and exhausted women, and I have now under my care a patient nearly thirty-five years old, who, in consequence of frequent abortion and protracted suckling, being exceedingly impoverished and feeble, has for the last 12 months, suffered from vicarious leucorrhœa. The regular menstrual period has been exactly observed, and although the discharge has been fully as abundant as the natural catamenia, and has lasted three or four days, it has never, till the last month been colored. Conception in these cases is not an improbable event, as in several females who have come under my notice, where the menstruation was colorless, pregnancy has occurred.

"In early life this vicarious leucorrhœa, if from its amount and periodical return, it is believed to be uterine and not merely vaginal, removes all impression of congenital defect or malformation. Nor, if the interval be free from excessive mucous discharge, is the health much deranged: a circumstance marking the difference between this form of vicarious menstruation, and chlorosis and amenorrhœa. It rarely happens that the uterine function is fully developed independently of medicine or change of air, although it is quite possible that, under favorable circumstances, perfect menstruation may almost spontaneously occur."

The treatment, as in the case of hemorrhage dependent on amenorrhœa, consists in remedies addressed to the uterus, such as purgatives, cupping or leeching, electricity, emmenagogues, and subsequently in the use of iron, aided by a nutritious and easily digested diet, exercise, and pure air. These measures will so far improve the blood and impart constitutional vigor, as sooner or later to induce healthy menstruation.

*Dysmenorrhœa* is the subject of the succeeding chapter, and is divided into a variety of species; as irritable or neuralgic, plethoric, inflammatory and congestive dysmenorrhœa, and amenorrhœa dependent on mechanical obstruction.—The pathology in each case being peculiar, a different plan of cure is demanded in each, and the great point is to arrive at a correct diagnosis in every instance. If that be clearly made out the course of treatment is easily determined. The neuralgic form must be treated by narcotics; the congestive and inflammatory by depletion, and so on. Acetate of ammonia has been highly recommended by a French writer in cases of painful menstruation, attended by morbid excitement of the female genital system, and Dr. Goddard, in a note to this work, mentions a case in which he witnessed the happy operation of the remedy in doses of a tablespoonful three times daily. It allayed the pain, which at previous menstrual periods had been very great. This remedy is said to be equally applicable in cases of profuse menstruation, or menorrhagia.

The fifth chapter is taken up with formulæ of remedies, which will be very convenient for practitioners. In the sixth chapter *menorrhagia*, or inordiate menstruation, is discussed. The author divides it into two forms, in the first of which the discharge is profuse, but unattended by uterine bleeding; and in the second with a profuse menstruation, there is attendant also loss of blood from the uterine arteries. The latter he considers under three varieties, namely; acute or active menorrhagia, occurring in the plethoric and robust; the passive or chronic, which is met with in delicate, exhausted, and hysterical females; and the congestive attendant upon middle or advanced life. These distinctions are valuable in a practical point of view, each variety demanding a peculiar treatment. Even in the first form, profuse menstruation, the plan of cure must vary with the habit of the patient, which may be plethoric and robust, or delicate, pallid and feeble. And in cases where hemorrhage exists it is of still more consequence, that the state of the system be carefully considered in the application of remedies. These modifications are pointed out by our author with great clearness. In order to be able to prescribe judiciously under such circumstances, Dr. A. justly remarks—

"It is right in every protracted hemorrhage from the uterus to *examine*. By this alone, and I allow that even then we frequently fail, can we expect to ascertain whether there be polypus, a sub-mucous tumour, or so much increased bulk as to render the existence of organic lesion highly probable. In the majority of examples of congestive menorrhagia, I believe that increased uterine bulk, fulness of the cervix, and openness of the os, constitute the whole of the diseased change."

The author speaks of injections into the uterus as efficient but at the same time as very hazardous remedies. He says:

"In conclusion, I think it right to observe, that I have twice witnessed, in most extreme cases, the beneficial effects of injecting into the uterine cavity a small quantity of the *spirit of turpentine.* It will not be supposed, after what I have heretofore said, that I advise this procedure on slight grounds. I believe such injections to be very certain but highly hazardous remedies, and they never ought to be employed except as *derniers resorts.* The uterus also has been injected with a small quantity of lead and alum in solution; and the narrator of the treatment says: 'the remedy is a dangerous one, for in two instances it was followed by vomiting, uterine inflammation, and death.'"

These remarks are followed by a case of so impressive a character that we cannot refrain from subjoining it:

"I first employed the turpentine in a menorrhagia where every previous remedy had proved ineffectual. The case is as follows:

"Mrs. G., forty-five years of age, and habitually intemperate, requested me to give her some medicine to prevent hemorrhage from the womb. She was large, and rather bloated, but still capable of great exertion. She was married, was the mother of several living children, and had miscarried a few months previously. I remonstrated with her on the excesses to which she acknowledged she was prone, and fully explained to her, that they were the source of the bleedings. The uterus was large and soft, and the cervix was full and flabby: but although the os was sufficiently patulous to permit of the entrance of the finger, I could not detect further structural change. An examination by the rectum was also made. She lived in my immediate neighborhood, and as I had frequent opportunities of seeing her, she adopted for a time the prescribed plan and diet. By purging during the intervals, and especially before the period, the losses were for a few months greatly diminished. At length she thought herself so well, as to be no longer under the necessity to adopt any plan which curtailed her usual indulgences. I lost sight of her for some months, and I know during the interval, that highly seasoned food, and large quantities of ale and wine were daily taken. One evening, I was requested in haste to visit her, and I found her almost dead from uterine bleeding. Her husband informed me, that since my last attendance, she had very frequently lost large quantities of blood, and he had thought that on several occasions she must have died, but that hitherto she had always slowly rallied. Brandy and ammonia and ergot, restored animation, but she had not said many words before a fresh gush again induced alarming syncope. Cold water was dashed over the face, ammonia was applied to her nostrils by a camel-hair pencil, and after a very lengthened fainting, she again rallied. On inquiry, I found the attack had already lasted two days; and it was evident that her powers were exhausted. Her voice was scarcely to be heard, the pulse was quick and feeble, and her breathing was very short, the countenance was livid and anxious; in fact, it seemed as though another gush would destroy her. Her medical attendant, Mr. Burton, plugged with sponge but ineffectually. On the instant I proposed to inject a small quantity of spirit of turpentine; and having procured a gum-elastic male catheter, and cut off its end, so that there was an open mouth, I introduced it through the os, which was very patulous, into the uterine cavity, and by a syringe I injected about two or three drachms of the spirit. Soon afterwards I plugged the vagina with tow. There was no further bleeding, but the pain was indescribably great, as though there were burning coals in the uterus and bladder. The evidences of hysteria seemed so clear, that I feared we must have taken away blood. Fomentations of poppy and conium applied very hot; camphor and laudanum, together with a purgative enema, allayed the intense suffering. In twenty-four hours I removed the tow, and there was no further bleeding. Menstruaton never returned, and from the continued and occasionally severe pain which followed the use of the turpentine, I suspect that adhesion of the sides of the uterine cavity resulted from the inflammation. Her former intemperate habits were soon resumed, and in less than a year she died. No inspection could be obtained."

And with this quotation we reluctantly close our notice of this excellent treatise. Our limits do not admit of a full analysis of its varied and instructive contents, and so we have confined our selections to the first few chapters. These, we feel very fully persuaded, will create in our readers a strong desire to see more of a work, in which complaints in every way so interesting to the practitioner have received so clear, practical, and full an illustration. It is for sale in this city by Mr. James Maxwell, through whom we are receiving from the publishers many similar favors.

